# *Scrophulariae* Radix: An Overview of Its Biological Activities and Nutraceutical and Pharmaceutical Applications

**DOI:** 10.3390/molecules26175250

**Published:** 2021-08-30

**Authors:** Hae-Jin Lee, Hae-Lim Kim, Dong-Ryung Lee, Bong-Keun Choi, Seung-Hwan Yang

**Affiliations:** 1 Department of Biotechnology, Chonnam National University, Yeosu 59626, Korea; haecutejin@naver.com (H.-J.L.); ics1357@naver.com (H.-L.K.); 2 NUON Co., Ltd., Jungwon-gu, Seongnam 13201, Gyunggi, Korea; drlee@nuon.kr (D.-R.L.); cbcbcbk@nuon.kr (B.-K.C.)

**Keywords:** *Scrophularia buergeriana*, *Scrophularia ningpoensis*, *Scrophulariae* Radix, biological activities, nutraceuticals, functional foods, in vitro study, in vivo study

## Abstract

*Scrophulariae* Radix (SR) has an important role as a medicinal plant, the roots of which are recorded used to cure fever, swelling, constipation, pharyngitis, laryngitis, neuritis, sore throat, rheumatism, and arthritis in Asia for more than two thousand years. In this paper, the studies published on *Scrophularia buergeriana* (SB) and *Scrophularia ningpoensis* (SN) in the latest 20 years were reviewed, and the biological activities of SB and SN were evaluated based on in vitro and in vivo studies. SB presented anti-inflammatory activities, immune-enhancing effects, bone disorder prevention activity, neuroprotective effect, anti-amnesic effect, and anti-allergic effect; SN showed a neuroprotective effect, anti-apoptotic effect, anti-amnesic effect, and anti-depressant effect; and SR exhibited an immune-enhancing effect and cardioprotective effects through in vitro and in vivo experiments. SB and SN are both known to exert neuroprotective and anti-amensice effects. This review investigated their applicability in the nutraceutical, functional foods, and pharmaceutical industries. Further studies, such as toxicological studies and clinical trials, on the efficacy and safety of SR, including SB and SN, need to be conducted.

## 1. Introduction

The genus *Scrophularia* consists of more than 300 different herbs, and *Scrophularia buergeriana* (SB) and *Scrophularia ningpoensis* (SN) are representative plants plants of this genus. SB is called “Hyun-sam” in Korea; it is a perennial plant with a strong fragrance that grows up to 1.8 m [1,2]. It is native to Korea, North China, and Japan, and it has an important role as a traditional medicinal herb. The SB root has been used to treat fever, swelling, constipation, pharyngitis, laryngitis, neuritis, sore throat, rheumatism, and arthritis, and it is also used for fire pursing, blood cooling, and toxin removal as oriental medicine [3,4,5]. SN is called “Xuan shen” in China, and it has been mainly used as a tea in traditional medicine. The SN root has been used to treat laryngitis, swelling, fever, constipation, and neuritis and it also used for immune enhancement [1,6]. In this paper, we reviewed the physiological activities of *Scrophulariae* Radix (SR), specifically, SB and SN. The physiological properties discussed in this review have been verified using in vitro and in vivo studies, and these results are the scientific basis for the development of health foods or therapeutics.

## 2. Phytochemicals in *Scrophulariae* Radix

Lee et al. [7] and Jeong et al. [8] reported that SB roots contain E-harpagoside, 8-O-E-p-methoxycinnamoyl-harpagide (MCA-Hg), E-p-methoxy-cinnamic acid (p-MCA), cinnamic acid, and angoroside C, which was set as the marker compound [9]. Kim et al. (2009, 2012) [3,10] and Shin et al. [4] reported that iridoid has been isolated from a wide variety of plants, including SB. Ren et al. [2] isolated 162 compounds from SN and, similar to Zhang et al., reported that iridoids, iridoid glycosides [11,12,13,14,15,16,17,18,19,20,21,22,23,24,25,26,27,28,29], phenolic acids, phenolic glycosides [30,31,32,33,34,35,36,37,38], flavonoids [39,40,41,42,43,44,45,46], terpenoids [47,48,49,50], organic acids [51,52], and other compounds [53,54,55,56,57] were identified in various studies. Gong et al. [58] suggested that iridoid glycosides and phenylpropanoid glycosides [59,60,61,62,63,64,65,66,67,68] were constituents of SR.

Most of the compounds isolated from SN were found in the roots and isolated by solvent fractionation. Iridoids and iridoid glycosides [11,12,13,14,15,16,17,18,19,20,21,22,23,24,25,26,27,28,29] are reported to have various biological activities including anti-inflammatory activity, immunomodulatory activity, anti-diabetic effect, and cardiovascular protection effect. In addition, it is known that flavonoids [39,40,41,42,43,44,45,46] isolated from SN have anti-hypertension effects and terpenoids [47,48,49,50] have anti-oxidative activity. Previous studies exhibited the anti-inflammation activity, anti-platelet aggregation activity, and anti-tumor biological activity of phenylpropanoid glycosides [59,60,61,62,63,64,65].

## 3. Evidence from In Vitro Studies

### 3.1. Anti-Inflammatory Effects

Shin et al. [4] reported that the 70% ethanol extract of SB (SBE, 10–80 μg/mL) regulated various inflammatory factors in raw 264.7 cells. Tumor necrosis factor (TNF)-α, IL-6, and matrix metalloproteinase (MMP)-9 are increased by lipopolysaccharide (LPS) induction, promoting an inflammatory response and increasing p65 phosphorylation. This inhibition of phosphorylation of p65 is considered a therapeutic target for asthma treatment. SBE inhibited TNF-α (SBE 40, 80 μg/mL: *p* < 0.01), IL-6 (SBE: *p* < 0.01), and MMP-9 (SBE 40, 80 μg/mL: *p* < 0.01) mRNA expression levels in LPS-induced Raw 264.7 cells. In addition, SBE significantly suppressed the expression of MMP-9 (SBE 10 μg/mL: *p* < 0.05, SBE 20–80 μg/mL: *p* < 0.01) and p65 phosphorylation (SBE 10, 40 μg/mL: *p* < 0.05, SBE 20, 80 μg/mL: *p* < 0.01). The mRNA expression levels of IL-6 and TNF-α in LPS-induced Raw 264.7 cells were reduced by SBE treatment with Bay11-7085 (NF-κB inhibitor).

### 3.2. Immune-Enhancing Effects

Kim et al. [10] demonstrated that SB water extract (0.01–1 mg/mL) with concanavalin A showed the immune-enhancing activity in MOLT-4 cells. The production of IL-2, IFN-g, and IL-2 induces a Th1-type cellular response, whereas IL-4, and IL-6 production increases Th2-type humoral immunity. SB water extract increased the production of IFN-γ (SB: NS, SB plus Con A 1 mg/mL: *p* < 0.05), IL-2 (SB 1 mg/mL, *p* < 0.05, SB plus Con A 0.1, 1 mg/mL: *p* < 0.01), and IL-4 (SB: NS, SB plus Con A 0.1, 1 mg/mL: *p* < 0.01) in MOLT-4 cells. Moreover, IgG (*p* < 0.05) production in the SNU 265 human B cell line increased with 1 mg/mL SB treatment. The cells treated with SB and SB plus IFN-γ showed increased IL-12 (SB: NS, SB plus IFN-γ 0.01 mg/mL: *p* < 0.05, SB plus IFN-γ 0.1, 1 mg/mL: *p* < 0.01) production and induced NO (SB 0.1, 1 mg/mL: *p* < 0.05, SB plus IFN-γ 0.1, 1 mg/mL: *p* < 0.01) level and iNOS (SB plus IFN-γ 0.1, 1 mg/mL) expression in mouse peritoneal macrophages.

Gong et al. [58] demonstrated that 85% ethanol extract of SR (ERS, 0.001–10 mg/mL) demonstrated the immune-enhancing effects in lymphocytes isolated from ICR mice spleen. The cAMP/cGMP ratio is known as an indicator of deficiency in immunity. In addition, MDA is an indicator that reflects oxidative stress, and SOD is an enzyme that converts superoxide radicals into molecular oxygen and hydrogen peroxide. ERS (1, 10 mg/mL: *p* < 0.01) treatment increased the cell viability in lymphocytes. In addition, ERS markedly decreased cAMP/cGMP (1 mg/mL: *p* < 0.05), IFN-γ/IL-10 (1 mg/mL: *p* < 0.01), and MDA content (1 mg/mL: *p* < 0.01) and increased the SOD content (1 mg/mL: *p* < 0.01) in lymphocytes isolated from ICR mice spleen.

### 3.3. Prevention of Bone Disorders

Nam et al. [5] reported that SBE (50–200 μg/mL) prevented the bone disorder in bone marrow macrophage. TRAP is an enzyme expressed in osteoclasts, and an increase in TRAP indicates mature and differentiated osteoclasts. SBE statistically (*p* < 0.001) suppressed TRAP-positive cell formation at 200 μg/mL concentration and had a resorption inhibition effect on mature osteoclasts. The resorption area (*p* < 0.001) was also observed to decrease after SB treatment.

### 3.4. Neuroprotective Effects

Lee et al. [7] demonstrated that SB 70% ethanol extract (SBE, 125–500 μg/mL) treatment showed the neuroprotective activity in SH-SY5Y cells. Acetylcholinesterase is an enzyme that the neurotransmitter acetylcholine, and increased activity of this enzyme affects the concentration of acetylcholine. SBE increased the cell viability (SBE: *p* < 0.01) in SH-SY5Y cells with glutamate-induced cell toxicity. Acetylcholinesterase activity (SBE 250 μg/mL: *p* < 0.05, SBE 500 μg/mL: *p* < 0.01) was decreased, and total glutathione content (SBE: *p* < 0.01) was increased in a dose-dependent manner. Glutamate-induced cell morphology changes and DNA fragmentation were measured using DAPI staining and TUNEL assays, and SBE was observed to reduce glutamate-induced fragmentation (SBE: *p* < 0.01).

Increased antioxidant enzyme activity is known to protect nerve cells by reducing oxidative neuronal damage. SOD-1, SOD-2, and GPx-1 are antioxidant enzymes, and when reduced, they promoted the expression of apoptosis factors such as Bcl-2-associated X (Bax), cleaved caspase-3, and cleaved poly (adenosine diphosphate (ADP)-ribose) polymerase (PARP). SBE treatment markedly increased SOD-1 (SBE: *p* < 0.01), SOD-2 (SBE: *p* < 0.05), and GPx-1 (SBE: *p* < 0.01) expression levels, but it decreased Bax (SBE: *p* < 0.01) protein, cleaved caspase-3 (SBE 125 μg/mL: *p* < 0.05, SBE 250, 500 μg/mL: *p* < 0.01), and cleaved PARP (SBE: *p* < 0.01) levels. SBE treatment also reduced the phosphorylation of p38 (SBE 250, 500 mg/mL: *p* < 0.05). In contrast, 500 μg/mL of SBE significantly (*p* < 0.01) increased B-cell lymphoma-2 (Bcl-2) expression levels.

Meng et al. [6] reported that *Scrophularia ningpoensis* water extract (RSAE, 6.25–50 μg/mL) demonstrated the neuroprotection effects in PC12 cells. RSAE differently increased according to pretreatment hour. Pretreatment RSAE for 4 h did not affect the cell viability and pretreatment RSAE for 8 h (12.5 μg/mL: *p* < 0.0.05), 16 h (12.5 μg/mL: *p* < 0.05), and 24 h (6.25–25 μg/mL: *p* < 0.00001) statistically increased the cell viability. LDH (RSAE 12.5 μg/mL: *p* < 0.0001), MDA (RSAE 12.5 μg/mL: *p* < 0.001), and NO (RSAE 12.5 μg/mL: *p* < 0.01) levels are decreased and SOD (RSAE 12.5 μg/mL: *p* < 0.05), CAT (RSAE 12.5 μg/mL: *p* < 0.05), and GSH-Px (RSAE 12.5 μg/mL: *p* < 0.0001) activities are recovered with RSAE in oxygen-glucose deprived and reperfusion (OGD/R)-induced PC12 cells. JC-1 red fluorescence for mitochondrial membrane potential change detection was significantly increased by treating with 12.5 μg/mL RSAE in PC12 cells. OGD/R treatment induced MMP destruction and significantly suppressed the red/green fluorescence ratio, but it significantly (RSAE 12.5 μg/mL: *p* < 0.0001) enhanced the red/green fluorescence ratio.

### 3.5. Anti-Apoptotic Effects

Shen et al. [1] demonstrated that *Scrophularia ningpoensis* water extract (RSN) presented the anti-apoptotic activity in HaCaT cells. RSN showed the IC50 value at 0.032 mg/mL concentration. TNF-a stimulation activates the NF-κB pathway and induces inflammation. In addition, ERK upstream of NF-κB affects cell proliferation and induces apoptosis. Pretreatment with 0.032 mg/mL RSN inhibited NF-κB translocation induced by TNF-α, which was observed using immunofluorescence staining of HaCaT cells. RSN decreased ERK phosphorylation, and ERK increased gradually with dose and time. RSN did not affect the cell cycle phase in G1/G0, S, and G2/M (at 0.016–0.064 mg/mL for 6–96 h) in HaCaT cells, and a tumor-preferred effect was not detected in Colo 38, SK-Mel-28, and MRI-221 cells. These results suggest that RSN regulates ERK and NF-κB signaling.

### 3.6. Anti-Allergic Effects

Kim et al. [3] reported that SBE (10–1000 μg/mL) showed anti-allergic activity in RBL-2H3 cells. SBE did not change the cell cytotoxicity and LDH releases at 1000 μg/mL concentration. SBE reduced the release of β-hexosaminidase (SBE 100 μg/mL: *p* < 0.05, SBE 1000 μg/mL: *p* < 0.001) and histamine (SBE 100, 1000 μg/mL: *p* < 0.05) in RBL-2H3 cells. The release of pro-inflammatory cytokines, TNF-α (*p* < 0.01) and IL-4 (*p* < 0.001), after antigen induction in RBL-2H3 cells was decreased after 1000 μg/mL SBE treatment. Furthermore, the cells treated with 100 and 1000 μg/mL SBE showed inhibition of ERK (SBE 100 μg/mL: *p* < 0.05, SBE 1000 μg/mL: *p* < 0.001) and p38 phosphorylation (SBE 10, 1000 μg/mL: *p* < 0.001).

## 4. Evidence from In Vivo Studies

### 4.1. Anti-Inflammatory Effects

Shin et al. [4] reported that SBE possesses anti-inflammatory activity. In the BALB/c asthma model induced by ovalbumin (OVA), SBE (20 and 40 mg/kg) was administered orally for 6 days. SBE administration significantly reduced eosinophils (SBE: *p* < 0.01), macrophages (SBE: *p* < 0.01), neutrophils (SBE 20 mg/kg: *p* < 0.05, SBE 40 mg/kg: *p* < 0.01), lymphocytes number (SBE 20 mg/kg: *p* < 0.05, SBE 40 mg/kg: *p* < 0.01), and total cells (SBE: *p* < 0.01) in bronchoalveolar lavage fluid (BALF) and also decreased airway hyper-responsiveness (SBE 20 mg/kg with methylcholine (MC) 30 mg/mL: *p* < 0.05, SBE 40 mg/kg with MC 20 mg/mL: *p* < 0.05, SBE 40 mg/kg with MC 30 mg/mL: *p* < 0.01).

The levels of pro-inflammatory cytokines, including IL-5 (SBE: *p* < 0.01), IL-13 (SBE 20 mg/kg: NS, SBE 40 mg/kg: *p* < 0.01), and IL-17 (SBE 20 mg/kg: *p* < 0.05, SBE 40 mg/kg: *p* < 0.01) in BALF and total IgE (SBE 20 mg/kg: *p* < 0.05, SBE 40 mg/kg: *p* < 0.01) and OVA-specific IgE (SBE 20 mg/kg: NS, SBE 40 mg/kg: *p* < 0.01) levels in the serum decreased after SBE treatment. SBE treatment significantly decreased the inflammatory index and mucus production index (SBE 20 mg/kg: *p* < 0.05, SBE 40 mg/kg: *p* < 0.01) in the lung tissue. In the asthma model induced by OVA, SBE administration decreased MMP-9 (SBE: *p* < 0.01) expression and p65 phosphorylation (SBE: *p* < 0.01).

These results suggest that SBE exhibits anti-inflammatory activity by inhibition of NF-κB phosphorylation, and it could be applied as an effective therapeutic agent against allergic asthma.

### 4.2. Anti-Amnesic Effects

Jeong et al. [8] reported that 70% ethanol extract of SB root (KD-501) possesses cognition-enhancing activity. Male ICR mice administered scopolamine, a substance known to cause short-term memory loss, exhibited induced amnesia. In these mice, KD-501 (3, 10, 30, 100, and 200 mg/kg) was administered either at once or for 15 days. Acute administration of KD-501 (10–200 mg/kg) (KD-501 10 mg/kg: *p* < 0.05, KD-501 30–200 mg/kg: *p* < 0.01), as well as treatment with 3–200 mg/kg KD-501 (KD-501 3, 10 mg/kg: *p* < 0.05, KD-501 30–200 mg/kg: *p* < 0.001) for 15 days significantly increased the step-through latency in the passive avoidance test. Acute and prolonged (for 15 days) treatment of 100 mg/kg KD-501 improved spatial memory ability by reducing escape latency in the Morris water maze test conducted for 4 days. The acetylcholinesterase activity of the cortex (KD-501: *p* < 0.05) and hippocampal (KD-501 100 mg/kg: *p* < 0.05, KD-501 200 mg/kg: *p* < 0.01) tissue was significantly increased by the acute administration of KD-501.

Acute treatment with 100 mg/kg KD-501 resulted in antioxidant activity by decreasing GSSG/total GSH (KD-501: *p* < 0.001 in hippocampus and increasing glutathione reductase (KD-501: *p* < 0.001 in Cortex, KD-501: *p* < 0.01 in hippocampus) and SOD (KD-501: *p* < 0.001 in Cortex, KD-501: *p* < 0.01 in hippocampus) activities in the cortex and hippocampal tissue of amnesic mice. The prolonged (for 15 days) oral treatment with 100 mg/kg KD-501 showed antioxidant activity by decreasing GSSG/total GSH (KD-501: *p* < 0.001) and increasing glutathione reductase (KD-501: *p* < 0.001) and SOD (KD-501: *p* < 0.001) activities in the cortex and hippocampus.

These results showed that KD-501 may be used for the prevention and therapeutics of Alzheimer’s disease.

Lee et al. [9,69] demonstrated that SBE has a neuroprotective effect on mice with memory impairment induced by scopolamine and β-amyloid. The mice were orally administered 30 and 100 mg/kg SBE for 28 days. The step-through latency (SBE: *p* < 0.05) in the passive avoidance test that decreased after scopolamine injection was significantly increased by treatment with 30 and 100 mg/kg SBE, and escape latency and swim distance were decreased in the Morris water maze test. Moreover, in the probe trial conducted on day 28, administration of SBE (30 and 100 mg/kg) significantly increased the crossing number (SBE: *p* < 0.01). The decreased acetylcholine (SBE 30 mg/kg: *p* < 0.05, SBE 100 mg/kg: *p* < 0.01) level was increased, while increased acetylcholinesterase (SBE 100 mg/kg: *p* < 0.01) activity was decreased in the hippocampus after SBE (30 and 100 mg/kg) administration.

Scopolamine injection reduced the BDNF expression levels and CREB phosphorylation. However, 100 mg/kg SBE treatment markedly increased the BDNF (SBE: *p* < 0.01) expression level, and 30 and 100 mg/kg SBE administration significantly increased CREB (SBE: *p* < 0.01) phosphorylation. Furthermore, SOD-1 (SBE: *p* < 0.01) and SOD-2 (SBE 100 mg/kg: *p* < 0.05) expression levels were increased and IL-1β (SBE 100 mg/kg: *p* < 0.01), IL-6 (SBE 30 mg/kg: *p* < 0.05, SBE 100 mg/kg: *p* < 0.01), and TNF-α (SBE 100 mg/kg: *p* < 0.01) gene expression levels were decreased in mice with SBE. The group injected with scopolamine showed increased expression levels of Bax, cleaved caspase-9, and cleaved PARP and decreased expression level of Bcl-2. The SBE treatment decreased Bax (SBE: *p* < 0.01), cleaved caspase-9 (SBE 100 mg/kg: *p* < 0.01), and cleaved PARP (SBE: *p* < 0.01) expression levels and increased Bcl-2 (SBE 100 mg/kg: *p* < 0.01) expression level.

The mice injected with β-amyloid showed decreased step-through latency in the passive avoidance test and increased escape latency and swim distance. SBE treatment (30 and 100 mg/kg) in these mice increased the step-through latency (SBE 30 mg/kg: *p* < 0.05, SBE 100 mg/kg: *p* < 0.01) and decreased escape latency and swim distance in the Morris water maze test. The crossing number (SBE 30 mg/kg: *p* < 0.05, SBE 100 mg/kg: *p* < 0.01) was also increased after SBE treatment. Glutathione reductase activity (SBE: *p* < 0.01) and SOD-1 (SBE 100 mg/kg: *p* < 0.01), SOD-2 (SBE 100 mg/kg: *p* < 0.01), and GPx-1 (SBE 100 mg/kg: *p* < 0.01) expression levels were decreased by β-amyloid injection and increased in the mice administered SBE. Furthermore, Bax (SBE: *p* < 0.01), cleaved caspase-9 (SBE: *p* < 0.01), and cleaved PARP (SBE: *p* < 0.01) levels were markedly decreased, and Bcl-2 (SBE 100 mg/kg: *p* < 0.01) expression level was increased after SBE treatment. The β-amyloid (SBE: *p* < 0.01) and phosphorylation of Tau (SBE: *p* < 0.01) were significantly decreased in the mice treated with SBE. According to the in vitro and in vivo studies, SBE improved spatial memory and cognitive ability by inhibiting cell apoptosis.

SBE indicated its potential for development as a health functional food for memory improvement and as a treatment for Alzheimer’s disease.

Meng et al. [6] reported that RSAE has a neuroprotective effect on the middle cerebral artery occlusion and reperfusion (MCAO/R) mouse model. Oral administration of RSAE (2.4 g/kg) for 7 days significantly decreased the brain water content (RSAE 2.4 g/kg: *p* < 0.05) and MDA (RSAE 2.4 g/kg: *p* < 0.01) and NO (RSAE 2.4 g/kg: *p* < 0.01) levels in the ischemic hemisphere. 2,3,5-Triphenyltetrazolium chloride (TTC) staining presented that the corrected infarct volume increased by MCAO/R operation was significantly decreased by RSAE (2.4 g/kg: *p* < 0.0001) administration. In the cortex and CA1 region, significantly increased neuronal damage due to MCAO/R was observed compared to the sham group. RSAE treatment significantly decreased neuronal damage by increasing neuron density in the ischemic cortex (RSAE 2.4 g/kg: *p* < 0.01) and hippocampus CA1 region (RSAE 2.4 g/kg: *p* < 0.01).

The MCAO/R group showed increased cell apoptosis by an increase in Bax-positive cells and a decrease in Bcl-2-positive cells. However, RSAE significantly reduced Bax (RSAE 2.4 g/kg: *p* < 0.05) expression and increased Bcl-2 (RSAE 2.4 g/kg: *p* < 0.0001) expression in focal cerebral ischemia and markedly decreased the phosphorylation of ERK1/2 (RSAE 2.4 g/kg: *p* < 0.05), p38 MAPK (RSAE 2.4 g/kg: *p* < 0.01), and JNK1/2 (RSAE 2.4 g/kg: NS). According to the in vitro and in vivo studies, RSAE inhibits apoptosis and exhibits neuroprotective effects by regulating the MAPK pathway.

These results suggest that RSAE could be a new therapeutic target for ischemic stroke patients.

### 4.3. Anti-Depressant Effects

Xu et al. [70] demonstrated that SN EtOAc extract (II) has an anti-depressive effect. Male ICR mice were orally treated with II (5, 10, 15, and 20 mg/kg) for 5 consecutive days, and the avoidance–escape test was performed. The extract of II (15 mg/kg: *p* < 0.01, 20 mg/kg: *p* < 0.005) remarkably decreased the number of escape failures and had an anti-depressive effect on the animal model.

These results suggest that II could be used for the treatment of depression.

### 4.4. Anti-Allergic Effects

Kim et al. [3] reported that SBE possesses anti-allergic activity. Dinitrofluorobenzene was used to induce the hypersensitivity reaction in BALB/c mice. SBE (10, 100, and 1000 μg/ear) was topically administered to the ear for 3 days.

SBE application reduced ear thickness (SBE 10 μg at 48 h, 72 h: *p* < 0.05, SBE 100 μg at 48 h: *p* < 0.01, SBE 100 μg at 72 h: *p* < 0.001, 1000 μg at 48, 72 h: *p* < 0.001) and weight (SBE 1000 μg at 48 h: *p* < 0.001, 100, 1000 μg at 72 h: *p* < 0.01) and decreased TNF-α (SBE 100 μg: *p* < 0.05, SBE 1000 μg: *p* < 0.01) and IL-4 (SBE 1000 μg: *p* < 0.01) levels in ear homogenates in a dose-dependent manner.

According to the previous in vitro and in vivo studies, SBE showed anti-allergic activity and may be effective in treating rhinitis and asthma.

### 4.5. Cardioprotective Effects

Huang et al. [71] demonstrated that the ethanolic extract of *Scrophularia ningpoensis* root (EERS) has a ventricular remodeling effect. Left ventricular remodeling was induced in rats orally administered with 60, 120, and 240 mg/kg EERS for 14 weeks. The ventricular remodeling group showed decreased systolic blood pressure (SBP), diastolic blood pressure (DBP), and mean arterial pressure (MAP) when compared with the sham-operated control. The ERRS-treated rats showed increased SBP (CAL plus 120 mg/kg EERS: *p* < 0.05), DBP (CAL plus 60, 120, and 240 mg/kg EERS: NS), and MAP (CAL plus 60, 120, and 240 mg/kg EERS: NS), and their increased heart rate (CAL 120 mg/kg: *p* < 0.05, CAL plus 60 mg/kg EERS: *p* < 0.01) was significantly reduced. The hemodynamic parameter left ventricular systolic pressure (CAL plus 120, 240 mg/kg EERS: *p* < 0.01) was remarkably increased by ERRS. Increased by left coronary artery ligation (CAL), cardiac weight indexes such as the left ventricular weight index (LVWI) (CAL plus 60, 240 mg/kg EERS: *p* < 0.05) and heart weight index (HWI) (CAL plus 60, 120, and 240 mg/kg EERS: *p* < 0.05) and cardiocyte cross-section area (CAL plus 60, 120, and 240 mg/kg EERS: *p* < 0.01) were decreased in the ERRS-treated group.

The ERRS-treated group showed that types I and III collagen volume (CAL plus 60, 120, and 240 mg/kg EERS: *p* < 0.01) and I/III collagen (CAL plus 60, 120, and 240 mg/kg EERS: *p* < 0.05) in the interstitial space were remarkably decreased. The interstitial collagen volume fraction (ICVF) (CAL plus 60, 120, and 240 mg/kg EERS: *p* < 0.01) and perivascular collagen volume fraction were also significantly reduced with ERRS treatment. The serum angiotensin II (Ang II) (CAL plus 120 mg/kg EERS: *p* < 0.05, CAL plus 240 mg/kg EERS: *p* < 0.01) concentration was decreased in the ERRS-treated rats. Endothelin (ET)-1 (CAL plus 60, 120, and 240 mg/kg EERS: *p* < 0.05), atrial natriuretic peptide (ANP) (CAL plus 60 mg/kg EERS: *p* < 0.05, CAL plus 120, 240 mg/kg EERS: *p* < 0.01), hydroxyproline (Hyp) (CAL plus 60, 120, and 240 mg/kg EERS: *p* < 0.01), MMP-2 (CAL plus 60, 120, and 240 mg/kg EERS: *p* < 0.01), and TNF-α (CAL plus 60, 240 mg/kg EERS: *p* < 0.01, CAL plus 120 mg/kg EERS: *p* < 0.05) concentrations were decreased by ERRS administration. The rats with ventricular remodeling that were administered ERRS showed a significant decrease in angiotensin-converting enzyme (ACE) (CAL plus 60, 120, and 240 mg/kg EERS: *p* < 0.01), ET-1 (CAL plus 60 mg/kg EERS: *p* < 0.01, CAL plus 120, 240 mg/kg EERS: *p* < 0.05), and ANP (Cal plus 240 mg/kg EERS: *p* < 0.05) mRNA expression levels.

These results suggest that ERRS exhibits preventive effects against cardiac fibrosis and attenuates ventricular remodeling. It could be used to treat early ventricular remodeling and heart failure.

Zhang et al. [72] showed that the active extract of *Scrophularia* Radix (ACRS) possesses ventricular remodeling inhibition effects. Spontaneously hypertensive (SHR) male rats were used for the experiment and orally treated with 70, 140, and 280 mg/kg ACRS for 21 weeks. The cardiac mass indexes LVWI (SHR with 70, 280 mg/kg ACRS: *p* < 0.01, SHR with 140 mg/kg ACRS: *p* < 0.05), HWI (SHR with 70, 140, and 280 mg/kg ACRS: *p* < 0.01), and ICVF (SHR with 70, 140, and 280 mg/kg ACRS: *p* < 0.01), perivascular collagen area (PVCA) (SHR with 70, 140, and 280 mg/kg ACRS: *p* < 0.01) ratio, collagen I (SHR with 70, 140, and 280 mg/kg ACRS: *p* < 0.01) and III (SHR with 140, 280 mg/kg ACRS: *p* < 0.01) volumes, I/III ratio (SHR with 140, 280 mg/kg ACRS: *p* < 0.05), serum norepinephrine (SHR with 140, 280 mg/kg ACRS: *p* < 0.05), myocardium Ang II (SHR with 140, 280 mg/kg ACRS: *p* < 0.05), and serum TNF-α levels were significantly decreased by ACRS treatment in the SHR rats.

The ACRS-treated SHR rats showed reduced collagen I (SHR with 70, 140, and 280 mg/kg ACRS: *p* < 0.01), TGF-β1 (SHR with 70, 140, and 280 mg/kg ACRS: *p* < 0.05), and ACE (SHR with 70, 140, and 280 mg/kg ACRS: *p* < 0.05) mRNA expression levels and decreased phosphorylation of p44/42 MAPK (SHR with 70 mg/kg ACRS: *p* < 0.05, SHR with 280 mg/kg: *p* < 0.01), SANP/JNK (SHR with 70, 140 mg/kg ACRS: *p* < 0.01, SHR with 280 mg/kg ACRS: *p* < 0.05), and p38 MAPK (SHR with 140, 280 mg/kg ACRS: *p* < 0.05). These results show that ACRS inhibited ventricular remodeling through MAPK pathway regulation, and it may be used to prevent and treat heart failure.

Gu et al. [73] showed that ERS possesses cardioprotective effects. Experimental ventricular remodeling was induced in the rats that were orally administered 8 (L) and 16 (H) g/kg ERS for 4 weeks. Cardiac weight indexes such as LVWI (ERS-L: NS, ERS-H: *p* < 0.05), HWI (ERS-L: NS, ERS-H: *p* < 0.05), myocardium Ang II (ERS-L: *p* < 0.05, ERS-H: *p* < 0.01), and Hyp (ERS-L and H: *p* < 0.01) decreased with the ERS treatment. To analyze the cardiocyte cross-section area (ERS-L and H: *p* < 0.01) by H&E staining, ERS administration was reduced similarly to that in the captopril group used as the positive control. In addition, PVCA (ERS-L and H: *p* < 0.01) and CVF (ERS-L and H: *p* < 0.01), which are increased by ventricular remodeling, were significantly decreased by the ERS treatment.

Subtypes I (ERS-L and H: *p* < 0.01) and III collagen contents (ERS-L: *p* < 0.05, ERS-H: *p* < 0.01) were significantly decreased and AT1R (ERS-L and H: *p* < 0.01), TNF-α (ERS-L and H: *p* < 0.01), and TGF-β1 (ERS-L and H: *p* < 0.01) mRNA expression levels were reduced in the myocardium by the ERS treatment.

These results suggest that ERS may be used for the treatment of myocardial infarction by decreasing the progression of left ventricular remodeling.

### 4.6. Immune-Enhancing Effects

Gong et al. [58] reported that ERS has immune-enhancing activity. The mice were administered 2 g/kg ERS intragastrically for 14 days, and the exterior signs of the mice with ERS were measured. Body weight (ERS: *p* < 0.01) was observed to significantly increase, and body temperature (ERS: *p* < 0.05), heart rate (ERS: *p* < 0.05), average speed (ERS: *p* < 0.01), and upright time (ERS: *p* < 0.01) were found to markedly decrease. Measurement of serum MDA and SOD levels and cAMP/cGMP suggested that ERS treatment decreased the MDA (ERS: *p* < 0.05) level and cAMP/cGMP (ERS: *p* < 0.05) and increased SOD (ERS: *p* < 0.05) level. The ERS-treated mice showed a statistical decrease in IL-6 (ERS: *p* < 0.01) expression level in the serum and Na^+^-K^+^ATP enzyme content (ERS: *p* < 0.01) in the liver homogenates. According to the in vitro and in vivo studies, ERS showed its immune regulation and antioxidant activities, and it can be used as dietary supplement for better health.

## 5. Conclusions

In this review, we summarized the biological effects of SB, SN, and SR based on the previous in vitro and in vivo studies. SB was administered orally at a minimum dose of 20 mg/kg to a maximum of 200 mg/kg (mice), SN was administered orally at a minimum dose of 5 mg/kg to a maximum of 2.4 g/kg (mice), and SR was orally administered at 2 g/kg (rat). SB showed anti-inflammation activity, immune-enhancing effects, bone disorder prevention activity, neuroprotective effect, anti-amnesic effect, and anti-allergic effect. SN exhibited neuroprotective effect, anti-apoptotic effect, anti-amnesic effect, cardioprotective effect, and anti-depressant effect; and SR showed immune-enhancing activity and cardioprotective effects. However, clinical trials need to be conducted to investigate the efficacy and safety of SR, including SB and SN.

As per studies published on SB, SN, and SR in the last 20 years, these plants have been processed using water or ethanol as a solvent. This is thought to reduce side effects when administered orally, and will help increase their applicability to various formulations when developing health functional foods or therapeutics in the future.

Taken together, we suggested the possibility of utilizing SB, SN, and SR for developing health functional foods or therapeutics for various applications on the basis of previously reported literature.

## Data Availability

Not applicable.
